# [Corrigendum] Relationship between changes in mitochondrial function and hippocampal neuronal apoptosis after recurrent convulsion during developmental stage

**DOI:** 10.3892/etm.2024.12429

**Published:** 2024-02-15

**Authors:** Yueying Liu, Jieru Chen, Meifang Jin, Zhenhong Li, Tian Tian, Lili Li, Hong Ni

Exp Ther Med 16:127–132, 2018; DOI: 10.3892/etm.2018.6147

Following the publication of the above article, the authors contacted the Editorial Office to explain that they inadvertently selected the same control GAPDH bands in Figs. 3 and 5 in their paper, both featured on p. 131. After having examined their original data, the authors identified all the data pertaining to a set of the alternative experiments, and wished to publish a corrigendum to present these figures correctly.

The corrected versions of Figs. 3 and 5 are shown on the next page. Note that the appearance of the incorrect control data in the original figures did not affect the overall conclusions reported in the paper, and the authors are grateful to the Editor of *Experimental and Therapeutic Medicine* for allowing them the opportunity to publish this corrigendum. The authors also apologize to the readership for any inconvenience caused.

## Figures and Tables

**Figure 3:**
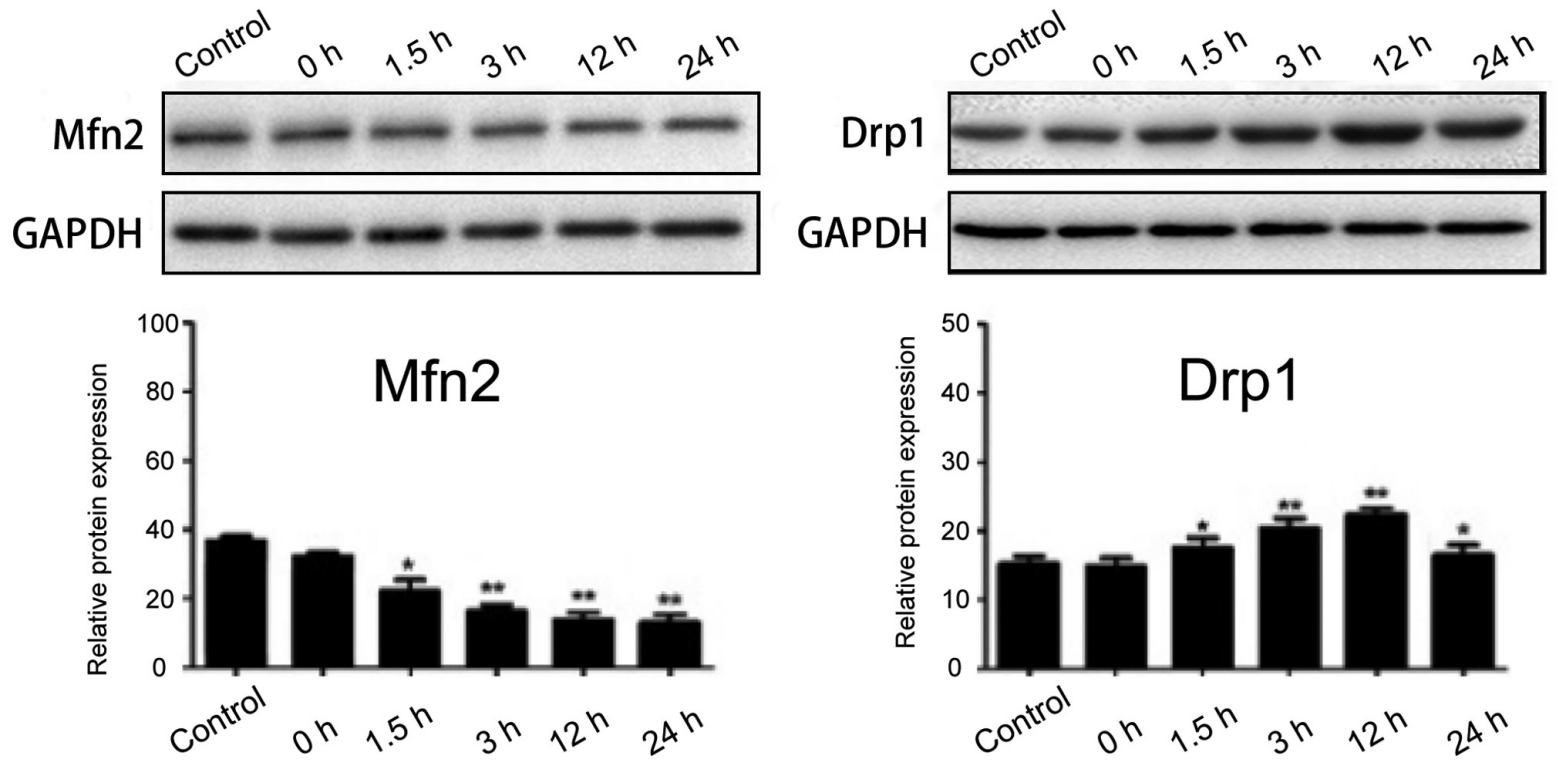
Expression of Mfn2 and Drpl proteins in each group. Compared with those in the control group, ^*^p<0.05, ^**^p<0.01 (n=3). Mfn2, mitochondrial fusion protein 2; Drp1, dynamin-related protein 1.

**Figure 5:**
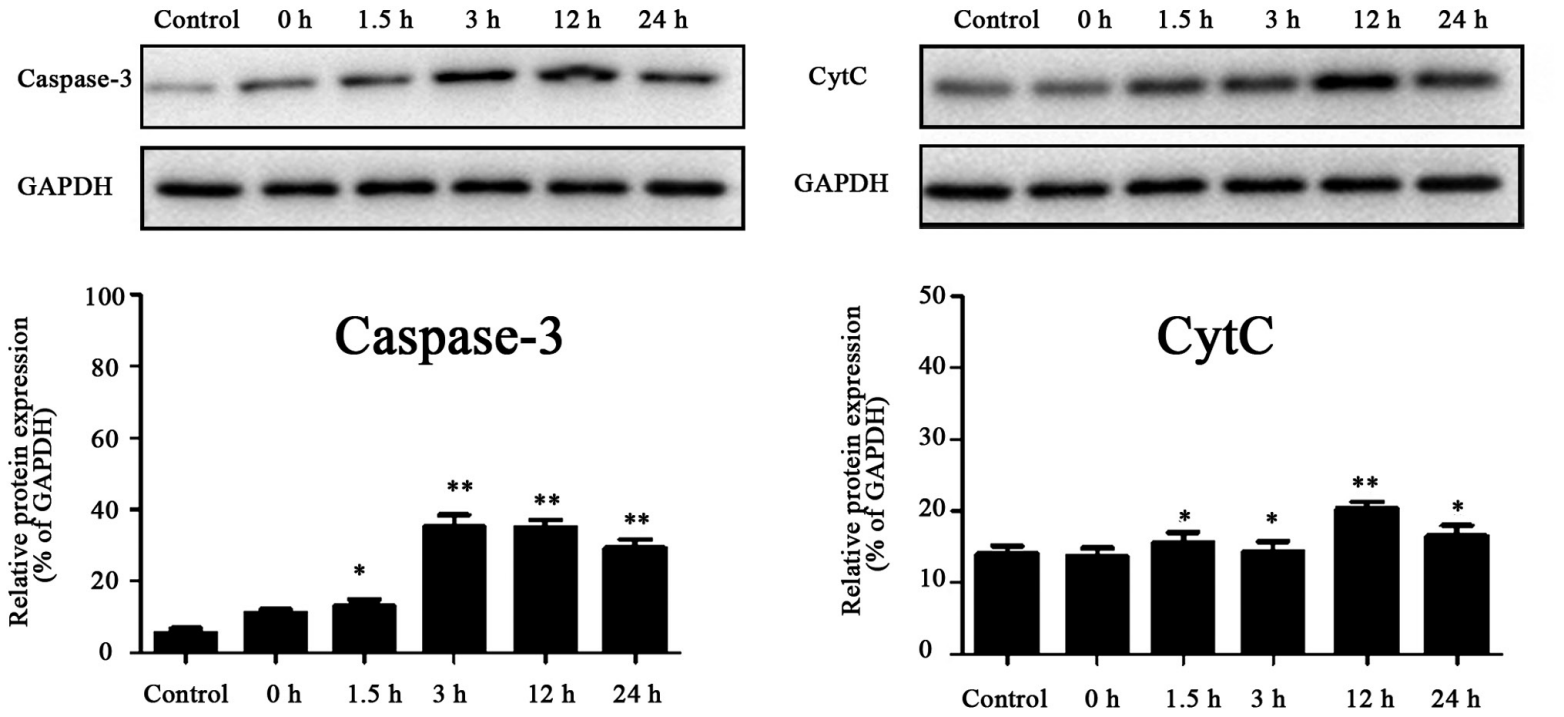
Expressions of caspase-3 and Cyt c proteins in each group. Compared with those in the control group, ^*^p<0.05, ^**^p<0.01 (n=3). Cyt c, cytochrome c.tumor necrosis factor-α.

